# Validation of virtual water phantom software for pre‐treatment verification of single‐isocenter multiple‐target stereotactic radiosurgery

**DOI:** 10.1002/acm2.13269

**Published:** 2021-05-24

**Authors:** Juan‐Francisco Calvo‐Ortega, Peter B. Greer, Marcelino Hermida‐López, Sandra Moragues‐Femenía, Coral Laosa‐Bello, Joan Casals‐Farran

**Affiliations:** ^1^ Servicio de Oncología Radioterápica Hospital Quirónsalud Barcelona Spain; ^2^ Servicio de Oncología Radioterápica Hospital Universitari Dexeus Barcelona Spain; ^3^ Department of Radiation Oncology Calvary Mater Newcastle Hospital Newcastle NSW 2298 Australia; ^4^ School of Mathematical and Physical Sciences University of Newcastle Newcastle NSW 2300 Australia; ^5^ Servei de Física i Protecció Radiològica Hospital Vall d'Hebron Barcelona Spain

**Keywords:** multiple‐target, SRS, virtual phantom

## Abstract

The aim of this study was to benchmark the accuracy of the VIrtual Phantom Epid dose Reconstruction (VIPER) software for pre‐treatment dosimetric verification of multiple‐target stereotactic radiosurgery (SRS). VIPER is an EPID‐based method to reconstruct a 3D dose distribution in a virtual phantom from in‐air portal images. Validation of the VIPER dose calculation was assessed using several MLC‐defined fields for a 6 MV photon beam. Central axis percent depth doses (PDDs) and output factors were measured with an ionization chamber in a water tank, while dose planes at a depth of 10 cm in a solid flat phantom were acquired with radiochromic films. The accuracy of VIPER for multiple‐target SRS plan verification was benchmarked against Monte Carlo simulations. Eighteen multiple‐target SRS plans designed with the Eclipse treatment planning system were mapped to a cylindrical water phantom. For each plan, the 3D dose distribution reconstructed by VIPER within the phantom was compared with the Monte Carlo simulation, using a 3D gamma analysis. Dose differences (VIPER vs. measurements) generally within 2% were found for the MLC‐defined fields, while film dosimetry revealed gamma passing rates (GPRs) ≥95% for a 3%/1 mm criteria. For the 18 multiple‐target SRS plans, average 3D GPRs greater than 93% and 98% for the 3%/2 mm and 5%/2 mm criteria, respectively. Our results validate the use of VIPER as a dosimetric verification tool for pre‐treatment QA of single‐isocenter multiple‐target SRS plans. The method requires no setup time on the linac and results in an accurate 3D characterization of the delivered dose.

## 1 INTRODUCTION

An increase in stereotactic radiosurgery (SRS) treatments for multiple (≥4) intracranial metastases has been described in the literature.^1^, ^2^ Traditionally, each lesion was treated using one or more isocenters, and the patient had to be shifted during a single session for repositioning according to the isocenter for each lesion.^3^, ^4^ A clear drawback of this approach is the longer time required to deliver a single fraction treatment with the corresponding discomfort of the patient. With the development of linac‐based intensity‐modulated radiotherapy (IMRT) and volumetric modulated arc therapy (VMAT), it is feasible to simultaneously treat multiple metastases using a single isocenter.^5^, ^6^, ^7^, ^8^, ^9^, ^10^

Patient‐specific quality assurance (PSQA) is a challenge in cases involving a large number of small, off‐isocenter, and widely spread‐out lesions. A detector with high spatial resolution and sufficient scanning spatial range to encompass all targets is need for this kind of PSQA. Large detection area (up to 40 × 40 cm^2^) and high spatial resolution (~0.3–0.4 mm) are available on current electronic portal imaging devices (EPID).^11^ Three‐dimensional (3D) dose reconstruction over the head volume is therefore feasible from EPID images, potentially enabling dose verification of single‐isocenter multiple‐target (SIMT) SRS plans.^12^ Ansbacher described a method for rapid evaluation of IMRT plans, using EPID images for reconstruction of the dose delivered to a virtual 3D cylindrical phantom.^13^ A similar method was used by the VIPER (VIrtual Phantom Epid dose Reconstruction) software developed at Calvary Mater Newcastle Hospital (CMNH) that has been used for remote EPID‐based external dosimetric auditing of IMRT and VMAT for clinical trials.^14^, ^15^ VIPER is not currently commercially available, but it can be made available on request for research purposes.

A dosimetric validation of the 3D dose reconstruction performed by VIPER is needed prior to be clinically used. However, accurate 3D measurements are extremely difficult to perform and only available at a few research centers. The Monte Carlo (MC) simulation is one of the most accurate methods in evaluating calculated dose distributions from other algorithms.^16^, ^17^, ^18^ MC simulation is often used to estimate dose distributions when experimental measurements are difficult or not possible to be performed. The PRIMO software (https://www.primoproject.net/) that permits MC simulations of Varian radiotherapy linacs in a user‐friendly manner, has been used in this study.^19^, ^20^

This study investigates the accuracy of the VIPER software to be used for pre‐treatment patient‐specific QA of single‐isocenter multiple‐target SRS plans. For small static MLC‐based fields, dose distributions computed by VIPER on flat water phantom were compared to 1‐D and 2‐D measurements in water tank and with film. Also, 3D dose distributions computed on a cylindrical water phantom for several SIMT SRS plans were compared with the corresponding 3D dose distributions reported by the PRIMO MC software.

## 2 MATERIALS AND METHODS

### 2.A Linac and VIPER software configuration

All measurements reported in this work were performed at Hospital Quirónsalud Barcelona (Spain) (HQB), which adopted fixed‐gantry intensity‐modulated radiosurgery in 2009 its standard procedure for cranial SRS. This was done after assessing the dosimetric advantages compared to a dynamic arc‐based SRS technique.^21^

Stereotactic radiosurgery is currently planned at HQB using a fixed‐gantry sliding window IMRT technique. SRS plans are calculated in the Eclipse treatment planning system (TPS) version 13.6 (Varian Medical Systems, Palo Alto, CA), using the analytical anisotropic algorithm (AAA), with a 1‐mm calculation grid size, and 6 MV photon beams from a Varian 2100 CD linac. A non‐coplanar beam arrangement (11–14 fields) with a single‐isocenter is always used. The linac is equipped with a Millennium 120 MLC and a PortalVision aS500 EPID with a sensitive area of 40 × 30 cm^2^ and a resolution of 512 × 384 pixels. This results in a pixel size of 0.784 mm when it is placed at a source‐detector‐distance (SDD) of 100 cm. The accuracy of the dose calculation performed by Eclipse for small lesions, and the targeting accuracy of single‐isocenter IMRT SRS for multiple lesions were previously investigated by our group.^22^, ^23^

VIPER is a software developed in MATLAB (The Mathworks, Natick, USA) at CMNH that allows EPID‐based 3D dose distribution reconstruction for combined IMRT fields and VMAT arcs of a plan in a “virtual” water phantom. Details of the algorithm to convert EPID images to dose have been previously detailed.^24^, ^25^ The method corrects for EPID sag and EPID support arm backscatter.

VIPER requires configuration for each linac used at each center. Two calibration plans (EPID and TPS calibration plans), and DICOM images of several virtual water phantoms are supplied by CMNH. The EPID calibration plan consists of fifteen static fields to be delivered to the EPID to determine EPID positioning and sag with gantry angle. The TPS calibration plan consists of a single 10 × 10 cm^2^ field with 100 monitor units (MUs), and gantry zero (Varian IEC 601‐2‐1 scale) with the isocenter at the center of each “virtual” phantom. The dose distributions derived from the local TPS (Eclipse, in this study) are used as calibration doses for the EPID‐to‐dose conversion model. The acquired EPID calibration images and TPS calculated doses are sent to CMNH to create a customized configuration file for each particular linac and TPS beam model.

In this study, the VIPER software (v. 3.10 beta, May 2019) was configured for 6 MV beams, for a dose rate of 600 MU per minute and for the EPID placed at a SDD of 100 cm. All deliveries were scheduled and managed using the Varian Aria version 13.6 record‐and‐verify system. All EPID images were acquired in‐air with no phantom or treatment couch present and using the integrated imaging mode. VIPER is provided with CT datasets of a “virtual” flat phantom of 20 cm height, 50 cm width, and 50 cm length (VFP20), and a “virtual” cylindrical phantom of 20 cm diameter and 40 cm length (VCP20). The VCP20 and VFP20 are named as “virtual” phantoms as no physical phantoms are irradiated and only in‐air EPID measurements are required by VIPER. The VFP20 phantom is intended for 2D single‐field analysis of each individual IMRT field of a plan, with normal incidence (zero gantry angle) and the isocenter placed at a depth of 10 cm. However, VIPER also performs the 3D dose reconstruction onto the VFP20 of a single field at gantry zero (Varian IEC 601‐2‐1 scale). Additionally, VIPER uses the VCP20 phantom for a 3D‐combined field analysis. This is the same principle as described by Ansbacher^13^ but using different dose calculation algorithms and depth‐dependent (5 cm intervals) EPID to dose conversion models combined with missing tissue and buildup corrections. Doses at other depths are interpolated and the 3D dose is rotated by the delivered gantry angle about the isocenter which is placed at the center of VCP20.

To perform a 3D VIPER‐based verification of a SRS plan, the plan has to be mapped in the Eclipse onto the VCP20 phantom and recalculated using the original MUs and fluences. The VCP20‐based plan is then delivered to the EPID, along with an open 10 × 10 cm^2^ field (100 MU) used by VIPER to calibrate the EPID signal to dose. In addition, an optional whole detector 40 × 30 cm^2^ field is used to determine the uniformity of the EPID. For all the plans included in this study, the 10 × 10 cm^2^ and 40 × 30 cm^2^ calibration images are acquired on each SRS plan verification. Once delivery is complete, the recorded EPID images (DICOM format), the RP DICOM plan file, and the RD DICOM dose file of the SRS plan to be verified are imported into the VIPER software. VIPER then computes the 3D dose distribution inside the VCP20 phantom for comparison with the TPS calculation using its 2D and 3D gamma analysis tools.

### 2.B Validation of the VIPER configuration

On‐site benchmarking of VIPER against measurement was performed by investigating the accuracy of the dose distributions reconstructed onto the VFP20 phantom for static 1 × 1, 2 x 2, and 3 x 3 cm^2^ fields. Field apertures were defined by the MLC with the jaws set at 10 x 10 cm^2^. One in‐air EPID image was acquired for each small field on three different days. Dose reconstruction was done for normal field incidence and a source‐to‐surface distance (SSD) of 90 cm. The number of MUs delivered to the EPID was 520, 450, and 430 MU for the 1 x 1, 2 x 2, and 3 x 3 cm^2^, respectively, giving a dose approximately 3 Gy at a depth of 10 cm on the central axis of the beam. These values were chosen to expose radiochromic films (see below) to a dose near the center of the calibrated range used in this study (0‐5 Gy).

Percentage depth dose (PDD) curves of the static 1 x 1, 2 x 2, and 3 x 3 cm^2^ MLC‐defined fields were acquired in a MP3 water phantom (PTW, Freiburg, Germany) for comparison with the PDDs calculated by VIPER. Measurements from the water surface up to a depth of 20 cm were acquired using a PinPoint ionization chamber (PTW 31014) and SSD of 90 cm. The diameter and length of the air cavity are 2 and 5 mm, respectively, giving a nominal active volume of 0.015 cm^3^. The chamber was setup in the MP3 water phantom with its stem parallel to the beam axis (axial orientation). Detector alignment with the beam central axis was attained using the CenterCheck tool of the PTW Mephysto mc2 v. 1.7.1 software (PTW, Freiburg, Germany). In addition, absolute doses given by VIPER at the central axis of the beam at depths of 1.5, 5, 10, and 15 cm were also compared to measurements. Measured and VIPER‐based PDDs were normalized at 10‐cm depth (mid‐VCP20 phantom) for comparison.

To derive absolute doses for these small MLC‐defined fields, output factors (OFs) relative to 10x10 cm^2^ were measured at a depth of 10 cm in the MP3 water phantom using the PinPoint ionization chamber in conjunction with a PTW Unidos electrometer. For these measurements, the detector was placed with its stem perpendicular to the beam axis (radial orientation), as recommended by the International Atomic Energy Agency Technical Report Series No 483 (IAEA‐TRS 483).^26^In addition, the derived output factors were corrected with the correction factors given in this report for small fields. PDD and OF ionization chamber‐based measurements were repeated on three different days.

For the static 1 x 1, 2 x 2, and 3 x 3 cm^2^ MLC‐defined fields, the two‐dimensional dose distributions reconstructed by VIPER at 10 cm depth of the VFP20 were compared to the respective measurements. GAFChromicTM EBT3 films (Ashland Inc., Wayne, NJ, USA) in a slab water equivalent phantom (RW3, PTW) were used. Three films per field size were exposed. Films were scanned 20 hr after exposure with an Epson Pro V750 flatbed scanner (Seiko Epson Corporation, Nagano, Japan) in transmission mode with a resolution of 150 dpi (0.2 mm/pixel) and 48‐bit RGB format. Film dosimetry was carried out using the web‐based application https://www.radiochromic.com (v. 3.2.2), which uses a multichannel algorithm to improve dose accuracy.^27^ The VIPER and film‐based measurements were compared in the Radiochromic.com software by performing a global gamma analysis with 3%/1 mm and 5%/1 mm criteria. The comparisons were performed within the 10% and 80% of maximum dose threshold to include and exclude the beam penumbra, respectively. Relative dose profiles through the beam central axis were extracted from the planar dose distributions to be compared.

**Fig. 1:**
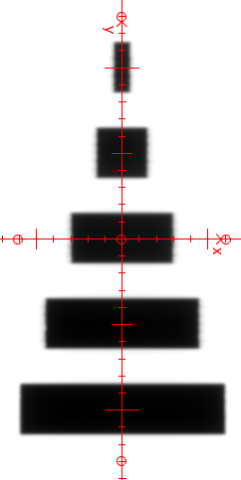
Aida test pattern.

Given the expressed advantage of a large measurement area for the VIPER method, some off‐axis apertures benchmarking has been included. The IMRT field known as the “Aida” pattern has been used.^28^ It represents a sequence of rectangle of decreasing width (from 12 to 1 cm) as shown in Figure 1. Dose computed by VIPER at the center of each aperture at 10‐cm depth of the VFP20 phantom were compared to the respective measured ones in the MP3 water phantom using the PinPoint ionization chamber in axial orientation.

**Fig. 2:**
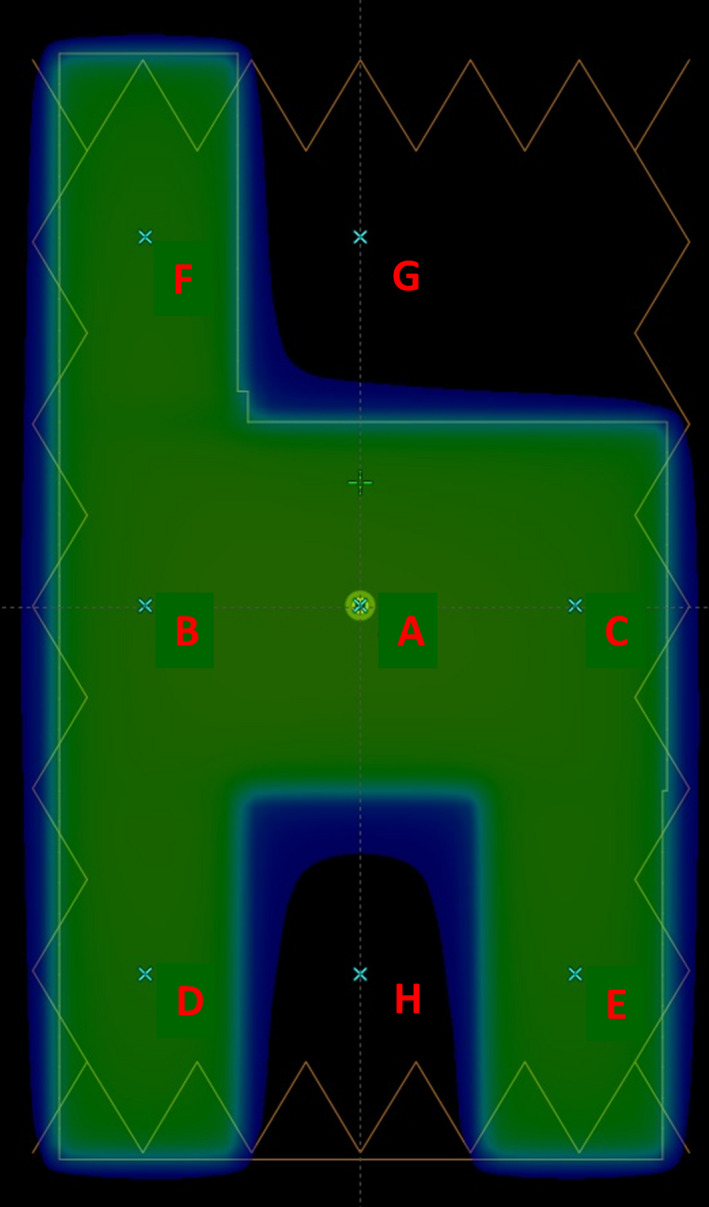
Chair test pattern.

Finally, verification of VIPER configuration against measurement was performed using the “chair” test (Figure 2) described by van Esch et al.^28^ This test is designed for quality control of IMRT using the sliding window technique. The chair test consists of a modulated field with three main regions defined in the irradiation pattern. The upper region allows separate evaluation of the impact of leaf transmission (point G) and dosimetric leaf gap (point F on the back of the chair). The central region is intended to create an area of homogeneous dose for accurate absolute dose verification (points A, B, and C). In the lower region, doses to the points D and E are influenced by both leaf transmission and dosimetric leaf gap, while point H is primarily affected by the leaf transmission.

In‐air EPID images were acquired for a “chair” test planned in Eclipse using the VFP20. A dose calculation was performed for 200 MU, resulting in approximately 7.5 Gy to point A at a depth of 10 cm. EPID images were acquired for this “chair” plan on three different days. Dose measurements using the PTW 31014 detector in axial orientation were performed in the MP3 water phantom at depths of 1.5, 5, 10, and 15 cm for the points shown in the Figure 2. The same cross‐ and in‐plane positions of these points were kept for all depths. Doses reconstructed by VIPER were compared with these measurements. Repeatability of the ionization chamber measurements was within 1%.

### 2.C Verification of the VIPER 3D dose reconstruction

The PRIMO MC software has been used in this study for verification of the full 3D dose distributions computed by VIPER for SIMT SRS plans. The accuracy of PRIMO (with the default simulation parameters) for the dose calculation of static 3DCRT beams was previously benchmarked by our group against the reference dosimetry dataset from IROC‐H (Imaging and Radiation Oncology Core–Houston). A dosimetric accuracy within 2.8% was found for 6 MV beams from the Varian Clinac 2100 CD with a Millennium 120 MLC used in the present study.^29^ Our team also validated PRIMO to be used for independent verification of IMRT SRS plans.^30^ SRS plans for single and multiple targets defined in a polystyrene phantom were simulated with PRIMO and dose planes were compared against radiochromic film measurements. Single targets consisting of spheres of 0.5, 1, 2, and 3 cm‐diameter directly outlined in the phantom CT images, while a set of three spheres of 1 cm‐diameter was outlined by mimicking a multiple target case. In addition, one brain metastasis (1 cm^3^) and one vestibular schwannoma (1 cm^3^) were mapped from two clinical cases to the phantom. All targets were located at the phantom with their centers at the film plane. GPRs ≥97% for a 5%/1mm global criteria, and local dose differences at the target centers within ±3.6% were found.

Eighteen SIMT SRS cases treated at HQB were retrospectively included in this study. Plans consisted of multiple non‐coplanar IMRT fields (11‐14 fields) treating multiple brain lesions (range: 2–35) with a single‐isocenter. Lesion volume ranged from 0.03 to 32.8 cm^3^ (median: 0.7 cm^3^). Lesion diameter ranged from 4 to 40 mm (median: 11 mm).

Each SIMT SRS plan was mapped in Eclipse to the VCP20 water phantom, and couch positions were set to 0 degrees (“verification plan”). The verification plan was re‐calculated in Eclipse with a grid size of 1 mm by keeping the same MUs, and then was delivered onto the EPID without any phantom. DynaLog files generated by the MLC controller during this delivery were retrieved, and the actual MLC segments were reconstructed using an in‐house code.^31^ For each verification plan, the DICOM RP file and the corresponding in‐air acquired EPID images were imported into the VIPER software to reconstruct the 3D dose distribution within the VCP20 (“VIPER plan”).

Finally, each verification plan was simulated within the VCP20 with the PRIMO MC software (v. 3.1.0.1772). A calculation voxel size of 1.2 mm × 1.2 mm × 1.0 mm was used. The simulation conditions used were described in a previous work.^29^ Simulation of each verification plan was done using the actual DynaLog file‐based MLC segments instead of using the planned MLC patterns (‛PRIMO plan’).

The dosimetric agreement between VIPER and PRIMO plans was assessed using the 3D gamma tool available in the PRIMO software. Global gamma analysis with the criteria of 3%/2 mm and 5%/2 mm were used in the present study. GPRs for both criteria were calculated within the ROIs of the VCP20 receiving at least 10%, 20%, 30%, 50%, 70%, 80%, and 90% of the maximum VIPER dose. A minimum GPR of 90% is considered as an acceptable level for the comparison, according to the AAPM Task Group No. 218.^32^

In addition, PRIMO allows computing the dose‐volume histogram (DVH) percentage of agreement (PA) for each ROI. The quantity PA was introduced by Rodriguez et al. as an indicator of the similarity of two DVHs, and it was shown to be more sensitive than the GPR to detect differences between dose distributions that are being compared.^33^ A PA value of 100% indicates a perfect DVH agreement. A minimum PA threshold of 95% is considered in this study as a good DVH agreement.

The median dose (D50) to a high‐dose volume (HDV) is another metric included for analysis to gain insight on the differences in absolute dose values. The 3D dose distributions reconstructed by VIPER were directly compared with the PRIMO calculations by the percentage difference in median dose to the high‐dose volume (%ΔHDVD50), as described by Olaciregui‐Ruiz et al.^34^ The selected HDV is the ROI80%, as the 80% isodose is a common prescription of the SRS treatments at HQB.

## 3 RESULTS

### 3.A Validation of the VIPER configuration

**Fig. 3:**
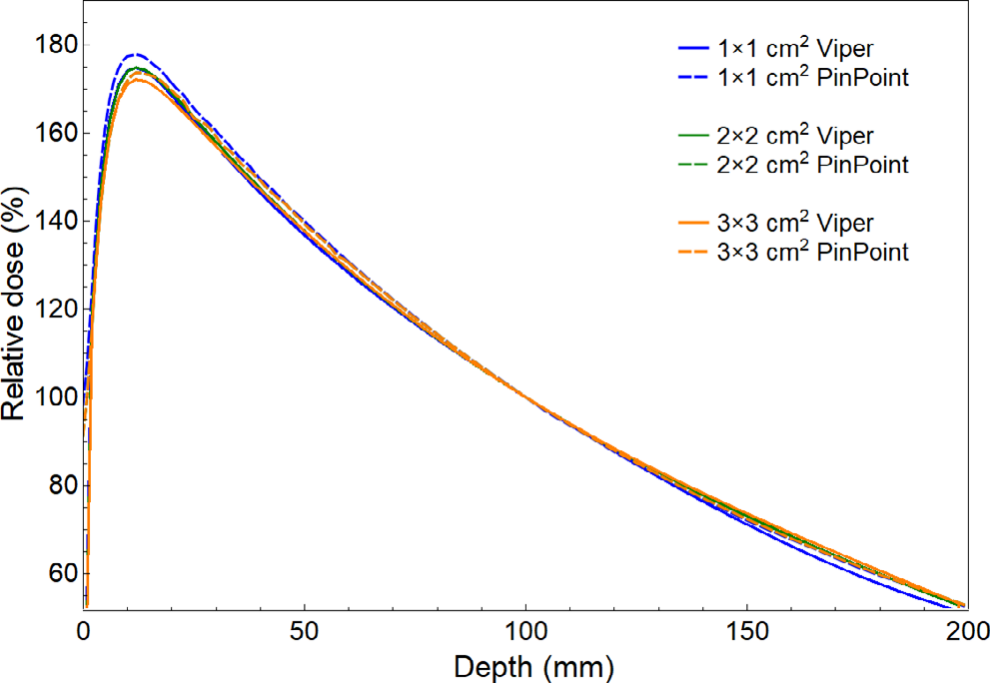
PDD comparison (VIPER vs. PinPoint‐based measured).

Figure 3 displays the PDDs calculated by VIPER and those measured with the PinPoint ionization chamber for the 1 x 1, 2 x 2, and 3 x 3 cm^2^ fields. PDD differences (VIPER vs. measured) at depths of 1.5, 5, 10, and 15 cm are reported in Table 1, with a maximum difference of 3.2% at 5 cm depth for the 1 x 1 cm^2^ field. Repeatability of the PDD values derived from VIPER and those measured with the ionization chamber collected on three different days was better than 0.05% and 0.2%, respectively. The percentage local differences (∆D) between the absolute doses given by VIPER and the respective absolute doses derived combining the measured OFs and PDDs are shown in Table 1, for the depths of 1.5, 5, 10, and 15 cm. The maximum difference was 3.2% at a depth of 15 cm for the 3 x 3 cm^2^ field. Repeatability of ∆D was better that 0.5% from data collected on three different days.

**Table 1:** VIPER vs. PinPoint‐based measurements: differences in PDD (∆PDD) and local dose differences (∆D) at several depths in water for three small fields.

Depth (cm)	1 × 1 cm^2^		2 × 2 cm^2^		3 × 3 cm^2^	
	∆PDD (%)	∆D (%)	∆PDD (%)	∆D (%)	∆PDD (%)	∆D (%)
1.5	−2.9	−0.9	0.5	1.3	−1.8	2.6
5	−3.2	−1.5	−1.6	−0.1	−1.6	1.1
10	0.0	0.6	0.0	1.0	0.0	1.7
15	−0.9	−1.1	0.7	1.8	1.3	3.2

**Fig. 4:**
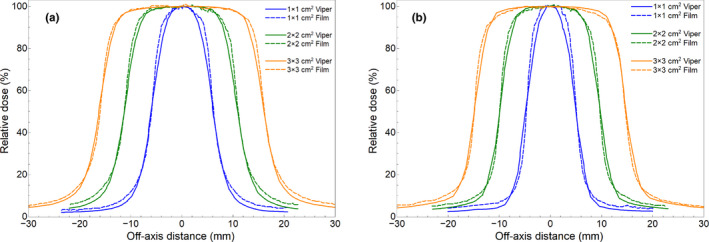
Comparison (VIPER vs. film‐based measured) of cross (a) and in‐plane (b) relative dose profiles at 10 cm‐depth.

Table 2 displays the OFs at a depth of 10 cm in water derived by VIPER from in‐air EPID images and those measured with the PinPoint ionization chamber. Repeatability of OF measurements was better that 0.5% from data collected on three different days. Differences of 1.8%, 1.0%, and 1.6% were found in the OFs for the 1 x 1, 2 x 2, and 3 x 3 cm^2^ fields, respectively. For off‐axis apertures, the Table 3 shows the local dose differences (VIPER vs. PinPoint‐based measurement) at the center of each rectangular aperture of the Aida test. Maximum difference of 0.8% was found for the smallest 1 x 3 cm^2^ aperture at 10 cm off‐axis distance from the central axis. Figure 4 shows the relative dose profiles at a depth of 10 cm in the VFP20 phantom provided by VIPER and those measured with film in RW3 for the 1 x 1, 2 x 2, and 3 x 3 cm^2^ fields.

**Table 2:** VIPER vs. PinPoint‐based measurements: differences in output factors (∆OF).

Metric	1 × 1 cm^2^	2 × 2 cm^2^	3 × 3 cm^2^
OF by VIPER	0.752 (0.003)	0.828 (0.002)	0.877 (0.001)
OF by PinPoint	0.739 (0.002)	0.820 (0.003)	0.863 (0.003)
∆OF	1.8%	1.0%	1.6%

Note

OF is defined at 10 cm‐depth in water and normalized to the 10 × 10 cm^2^ open field. Figures in parentheses are the standard deviation due to three measurements on different days.

**Table 3:** VIPER vs. PinPoint‐based measurements: local dose differences at the center of each aperture of the Aida pattern.

Field aperture	1 × 3 cm^2^	3 × 3 cm^2^	6 × 3 cm^2^	9 × 3 cm^2^	12 × 3 cm^2^
Off‐axis distance	−10 cm	−5 cm	0 cm	5 cm	10 cm
Dose difference	0.8%	0.1%	0.1%	0.5%	0.2%

Note

divisions each 1 cm are shown in the displayed cross‐hair.

Table 4 shows the differences (VIPER vs. measured) found in field size (FWHM) and penumbras along the cross‐ and in‐plane directions, as quantified from three film measurements per field for the 1 x 1, 2 x 2, and 3 x 3 cm^2^ fields. Cross‐plane direction coincides with the leaf motion direction. VIPER always overestimated the beam penumbra (up to ~1 mm). The differences between the field size values given by VIPER and film were within 0.4 mm. Figure 4 shows systematic underestimation (<2.5%) of the out‐of‐field dose by the VIPER software. The GPRs of the 2D global gamma evaluation between the VIPER‐reconstructed dose planes at 10 cm‐depth in the VFP20 phantom and the reference 2D dose distributions measured with film in RW3 were above 95% for all the static fields, for both global 3%/1 mm and 5%/1 mm criteria (Table 5).

**Table 4:** VIPER vs. film‐based measurements: differences in field sizes (∆FS) and beam penumbras (∆Pen) at 10‐cm depth in water.

Metric	1 × 1 cm^2^	2 × 2 cm^2^	3 × 3 cm^2^
∆FS (mm) / cross‐plane	0.0 (0.1)	0.1 (0.1)	0.0 (0.2)
∆FS (mm) / in‐plane	0.1 (0.1)	0.1 (0.1)	0.4 (0.0)
∆Pen (mm) / cross‐plane	0.7 (0.1)	0.5 (0.1)	0.7 (0.2)
∆Pen (mm) / in‐plane	1.1 (0.2)	1.0 (0.1)	0.9 (0.2)

Note

Figures in parentheses are the standard deviation due to three measurements on different days.

**Table 5:** VIPER vs. film‐based measurements: 2D gamma passing rates (in %) for three small fields at 10‐cm depth in water.

Global Criteria:	1 × 1 cm^2^	2 × 2 cm^2^	3 × 3 cm^2^
3%/1 mm 10%	99.4 (0.6)	100 (0.1)	98.9 (1.1)
3%/1 mm 80%	97.0 (3.5)	99.9 (0.1)	97.6 (2.4)
5%/1 mm 10%	100.0 (0.2)	100.0 (0.0)	100.0 (0.0)
5%/1 mm 80%	99.8 (0.3)	100.0 (0.0)	100.0 (0.0)

Note

3%/1 mm and 5%/1 mm criteria, with two low‐dose thresholds (10% and 80%), are used. Figures in parentheses are the standard deviation due to three film measurements.

Table 6 shows the dose differences for the eight selected points of the ‛chair’ test in the VFP20 phantom. Small local dose differences (<2%) were found for the six in‐chair points (A, B, C, D, E, and F) between depths of 5 and 15 cm, while discrepancies up to 4% were obtained at the depth of maximum dose (1.5 cm). VIPER always underestimated doses in the MLC transmission areas up to 4.1% (points G and H). Over three measurements, the standard deviation of the dose points reported by VIPER was better than 0.5% for the in‐chair points and ~1.0% for the points G and H, while repeatability of the ionization chamber dose point measurements was within 0.5%.

**Table 6:** VIPER vs. Pinpoint‐based measurements: local dose differences at several depths in water for the selected points of the “chair” test.

Point	1.5‐cm depth	5‐cm depth	10‐cm depth	15‐cm depth
A	2.8%	0.4%	0.4%	0.2%
B	2.8%	−0.1%	0.4%	0.1%
C	2.9%	0.9%	1.3%	0.9%
D	3.8%	1.3%	1.8%	2.2%
E	3.5%	1.5%	1.8%	1.4%
F	2.2%	0.4%	1.9%	0.4%
G	−1.7%	−2.1%	−1.7%	−2.8%
H	−1.7%	−2.6%	−3.0%	−4.1%

Note

Grey shadow indicates differences below 2%.

### 3.B Verification of the 3D dose VIPER reconstruction

**Fig. 5:**
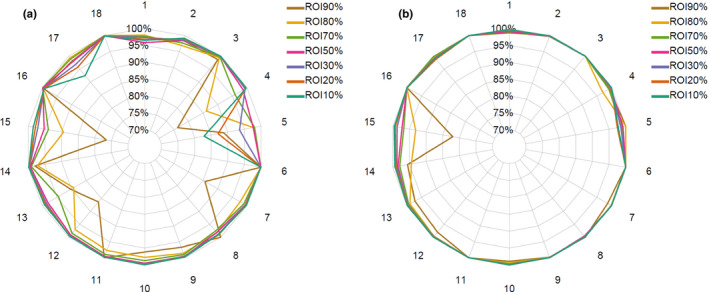
3D Gamma passing rates (GPRs) for 18 SIMRT SRS plans with (a) 3%/2 mm and (b) 5%/2 mm criteria.

Figure 5 shows the 3D global GPRs after comparing the reconstructed 3D dose distributions obtained from VIPER with the reference 3D dose distributions calculated by PRIMO, for the 18 SIMT SRS plans analyzed. For the 3%/2 mm criteria (Table 7), average GPRs over the 18 cases were greater than 93.7% in all ROIs, with GPRs ranging from 76.3% to 100%. GPR was lower than 90% in 10 cases. For the 5%/2 mm criteria (Table 7), average GPRs greater than 98.2% were reached in all ROIs, with GPRs ranging from 82.0% to 100%. GPR was lower than 90% just in one case and it was noticed in the ROI_90%_ (GPR of 82.0%). GPR values for each ROI and case are plotted in Figure 5, and no failure trend was observed in any ROI. Tables with all values are available as supplemental data. For each ROI, the PA value averaged over the 18 cases was always greater than 95%, except for the ROI_10%_ with a PA of 94.8% (Table 7). The average value of the %ΔHDV_D50_ metric was 2.0% (range: 0.0% to 4.7%). %ΔHDV_D50_ was within 3% in 15 of the 18 cases.

**Table 7:** VIPER vs. PRIMO: comparison of the 3D dose reconstructed by VIPER to that simulated with PRIMO for 18 SIMT SRS plans.

ROI	Criteria: 3%/2 mm		Criteria: 5%/2 mm		Mean PA
	Mean GPR (%)	# <90%	Mean GPR (%)	# <90%	(%)
ROI_90%_	93.7 (7.9)	5	98.2 (4.2)	1	97.5 (1.4)
ROI_80%_	96.7 (4.0)	3	98.9 (2.0)	0	97.8 (1.3)
ROI_70%_	98.2 (1.8)	0	99.4 (0.9)	0	97.7 (1.2)
ROI_50%_	98.7 (1.4)	0	99.5 (0.7)	0	97.5 (1.3)
ROI_30%_	98.7 (1.6)	0	99.6 (0.5)	0	96.8 (2.0)
ROI_20%_	98.4 (3.1)	1	99.6 (0.6)	0	96.2 (2.5)
ROI_10%_	98.1 (4.2)	1	99.7 (0.5)	0	94.8 (3.3)

Note

Mean gamma passing rates (GPRs) and mean DVH percentage agreement (PA) values are shown for seven regions of interest (ROIs). The values in brackets give the ranges of the results. The number of cases with GPR below 90% (#<90%) is also shown.

The average PRIMO simulation time was 161.7 min (range: 86.4–386.2 min), and the statistical uncertainty (k = 2) of the dose estimated by PRIMO ranged from 1.6% to 3.7% (average: 2.3%).

## 4 DISCUSSION

To date, the only true 3D dosimeters with high spatial resolution for multiple‐target SRS PSQA are polymer gel dosimeters. However, the implementation of the gel‐based method in a SRS program is impractical for the daily clinical practice. A more efficient method to provide 3D dose reconstruction over the head volume is needed for the dose verification of SIMT cranial SRS plans. In this study, we have evaluated the accuracy of the VIPER software to perform 3D EPID dosimetry focused in pre‐treatment patient QA of SIMT SRS plans.

The accuracy of the VIPER software to reconstruct dose within a virtual water phantom was previously investigated by Miri et al.^35^ For 36 IMRT fields, they compared the 2D dose distributions reconstructed by VIPER at a depth of 10 cm with the dose measured by a 2D diode array. The model used by VIPER was validated with mean gamma passing rates >98% for 3%/3 mm criteria. However, a 3D evaluation was not performed as a 2D detector was used.

The accuracy of VIPER was investigated in this study using three small static fields (1 x 1, 2 x 2, and 3 x 3 cm^2^) and the “Aida” and “chair” tests for IMRT delivery. Tables 1, 2, 3, 4, 5, 6 are shown quantitative validation of VIPER dose calculation against dose measurements performed in water and RW3 phantoms, for small static MLC‐based fields and the two well‐established Aida and chair patterns for IMRT delivery. The reported differences were within ±3% and 1 mm for the majority of evaluated points, reflecting an excellent performance of VIPER for this scenario. Assessing VIPER as a “treatment planning system”, these differences are within the tolerances described in the MPPG 5.a document.^36^

Figure 4 shows the systematic VIPER underestimation of the shoulder width of the central cross‐plane relative dose profiles. It can be attributable to the worse spatial resolution of the EPID (0.784 mm/pixel), in comparison to the film detector (0.2 mm/pixel). However, small discrepancies (<1.1 mm) in the penumbra of the small static 1 x 1, 2 x 2, and 3 x 3 cm^2^ fields were found between the VIPER and the film‐based methods (Table 4).

Due to its high spatial resolution, radiochromic film is a very suitable detector for lateral profile measurements in small fields (IAEA TRS‐483). Close agreement (≤0.5 mm) has been described between the profiles of small fields measured with EBT3 film and those acquired with a PinPoint detector.^37^ Therefore, the penumbra differences (≤1.1 mm) observed in Figures 4 cannot be attributed to a film limitation, and seems due to the modelling of the VIPER software, which has not been specifically optimized for small fields.^15^ Hence, although there is margin for improvement in the VIPER modeling of the penumbra, it is still adequate for the purposes of this work.

From Table 6, VIPER underestimated doses in the high MLC transmission regions of the chair test which is a known issue with EPID response but does not affect gamma pass rates significantly.^38^, ^39^

A potential source of inaccuracy of this study is that the point doses measured using the PinPoint ionization chamber were made in the very large MP3 water phantom (73 cm × 52 cm × 64 cm) in contrast to the size of the VFP20 phantom (20 cm × 20 cm × 40 cm) used by VIPER for dose reconstruction, such that difference in scatter could be an issue for dose comparison (VIPER vs measured). However, the excess of scatter due to the larger water phantom MP3 was estimated in Eclipse and it resulted in a negligible error of less than 0.2%.

It is well known that hardening of the photon energy spectrum of small fields occurs at any point on the beam central axis with decreasing field size.^26^ This fact is revealed by the PDD values measured with the PinPoint chamber, but not by the PDDs values given by VIPER (Table 1). We think that this issue could be solved by adjusting the current parameters of the depth‐dependent scatter EPID kernel of VIPER to improve the agreement for depth doses on the central axis for small fields. The accuracy of VIPER was investigated for auditing IMRT/VMAT plans in the Virtual Epid Standard Phantom Audit (VESPA) project.^14^ However, the EPID‐to‐dose conversion performed by VIPER was not specifically developed for small field dosimetry, as has been performed in some back‐projection portal dosimetry models.^40^

The accuracy of VIPER for 3D dose reconstruction was assessed using a gamma index analysis for 18 IMRT SRS plans by using PRIMO MC simulations as reference. As Miften et al stated, there is a need to consider both the spatial and dosimetric uncertainties when comparing dose distributions to determinate if the reference and evaluated dose distributions (PRIMO and VIPER, resp. in our study) agree to within the limits that are clinically relevant.^32^

A typical 3% limit is taken as the acceptance criterion for the dose differences during IMRT PSQA.^32^ To use this criterion of 3% presumes that the uncertainty in PRIMO itself is significantly less than this value. However, we believe it is appropriate to expand the 3% gamma dose criterion to 5% to account for the statistical uncertainty (~2%) of the PRIMO dose distributions.^41^ For instance, this 5% criterion has been established for stereotactic treatment verification in the Report 25 of the Netherlands Commission on Radiation Dosimetry (NCS 25).^42^

The spatial analog to the dose difference is the distance‐to‐agreement (DTA) metric. As the dose distribution measurements have some spatial uncertainty, the DTA criterion can be partly defined based on the measurement error. The positional accuracy of the EPID used in our study has been evaluated in 0.6 mm. The dose calculation has a non‐zero spatial error. We think that it is at least comparable to the voxel size of the PRIMO simulations (1.2 mm × 1.2 mm × 1.0 mm). In other words, the PRIMO dose voxel has not a resolution of less than 1 mm such that the dose calculation algorithm itself contains some uncertainty in placement of dose within the voxel. Therefore, although SRS delivery accuracy is aim for <1 mm, we have chosen a distance to agreement of 2 mm for the gamma analysis to take into account experimental and calculation uncertainties.

In the context of PTV margins used for treatment, Bossuyt et al described to use the CTV‐PTV margin as DTA for transit in‐vivo PSQA based on EPID measurements.^43^ The 2‐mm DTA used in the present study coincides with the CTV‐PTV margin implemented in our SIMT SRS policy.^44^ So, a successful GPR using 2‐mm DTA is compatible with an adequate dosimetric coverage of the lesions.

**Fig. 6:**
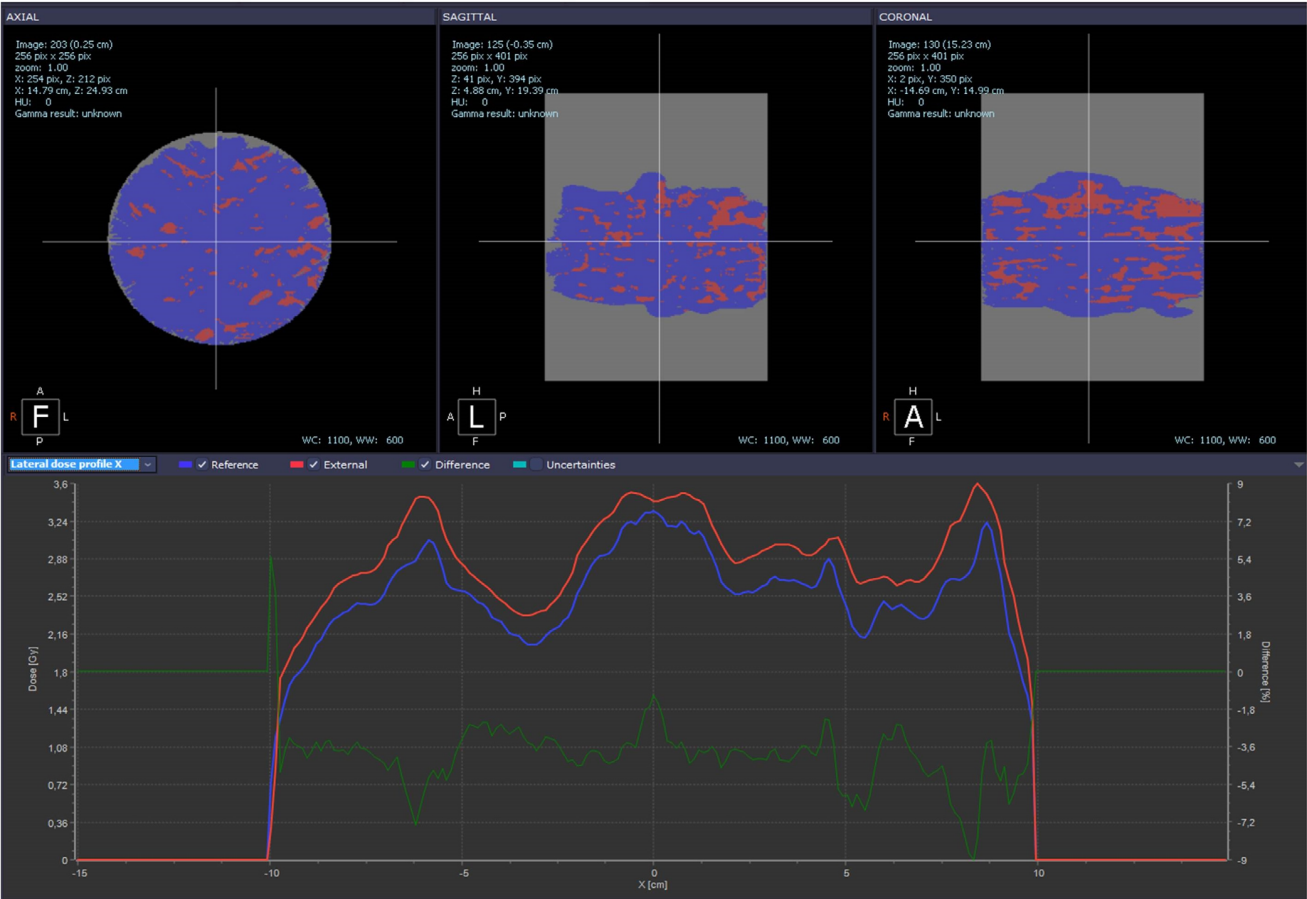
Gamma index maps for 3%/2 mm at the central axial, sagittal and frontal slices of the VCP20, for case 5. Comparison of dose profiles through the R/L direction shows that differences are mainly due to dose differences rather than profiles misalignment (red: VIPER; blue: PRIMO; green: dose difference).

Our results indicate an excellent dosimetric agreement between the 3D dose distributions reconstructed by VIPER and PRIMO on the VCP20 phantom. Over the 18 SIMT SRS plans, the 3%/2 mm and 5%/2 mm GPR average values ranged from 93.7% to 98.7% and 98.2% to 99.7%, respectively, along the seven ROIs segmented for each plan in the VCP20 (Table 7). Most of the failures were noticed with the 3%/2 mm criteria. After inspection of the gamma index map for each case, we found that gamma index values >1 were due to differences in the dose peaks and low‐dose tails of the profiles (VIPER vs. PRIMO, see Fig. 6), rather than geometrical misalignments. It was also noted that the failure rate was higher for the ROI90% as the number of voxels included was lower than the other ROIs, such that a few failing voxels will give a low gamma passing rate. So, the PA metric can aid to assess the VIPER vs. PRIMO agreement. High values (≥95%) of PA were reported in all ROI90% regions. However, the number of cases with GPR below 90% decreased from 10 to 1 when the dose criterion changed from 3% to 5%. A closer look to this single failing case revealed a PA value of 96.5% for the ROI (ROI_90%_) where GPR was less than 90%. Thus, our results indicate that VIPER 3D dose reconstruction is accurate within 5% and 2 mm for SIMT SRS plans.

As expected, changing the criterion from 3% to 5% improved the GPR, with no additional margin for uncertainties in the patient dose delivery whether a goal of 5% is required. However, a single statement about accuracy requirements, i.e., 5% in radiotherapy, is an oversimplification. The accuracy requirements are dependent on both technological considerations as well as biological and clinical concerns. For instance, the Imaging and Radiation Oncology Core (IROC) performs SRS audit using a 5%/3 mm criteria for 2D gamma analysis with their intracranial SRS phantom (Kry, S. 2020; personal communication). The spatial target localization is the key issue for a successful SRS treatment. The 5% accuracy found in our study for the VIPER software is compatible with the 5%–6% criterion established in the NCS 25 guideline for stereotactic treatment verification.^42^ The 5% dose criterion has been also used for analysis of the accuracy of VMAT‐based SIMT SRS using measurement‐based 3D dose reconstructions.^4^, ^45^, ^46^

In contrast to our derived 5% accuracy level for the VIPER software, some authors used tighter dose criterion for the gamma analysis. For example, Ahmed et al investigated the accuracy of a commercial hybrid volumetric dose verification system for SIMT cranial SRS.^12^ For several SRS plans, they compared the dose distributions reconstructed by this system inside a cylindrical phantom with planar doses measured with film orientated inside this phantom at several angles. While it was not a full 3D dose verification, the authors obtained excellent 2D GPRs (>96%) using a 3%/1 mm criteria with a dose threshold of 10%. The TG‐218 recommends tolerance and action level limits for a 10% dose threshold. If we look into our 3D GPRs for the 10%‐ROI very good agreement (98.1% ± 4.2%) is achieved for our SIMT SRS plans when 3%/2 mm criteria were used.

There is scarce literature about virtual phantom 3D dose reconstruction using in‐air EPID measurements. Olaciregui‐Ruiz et al.^34^ described the use of a research version of the iViewDose system (Elekta AB, Stockholm, Sweden), that allows performance of non‐transit 3D EPID dosimetry within a virtual water phantom. While SRS plans were included in that study, no specific multiple‐target verification was done. Alhazmi et al.^47^ developed an in‐house EPID‐based algorithm to reconstruct a 3D dose distribution as imparted to a virtual cylindrical water phantom to be used for plan‐specific pre‐treatment dosimetric verification for IMRT and VMAT plans, but analysis of SRS plans was not described. Both studies concluded that non‐transit 3D EPID dosimetry can be readily used for pre‐treatment PSQA of IMRT, eliminating the need of phantom positioning. Our study agrees with this conclusion, with the novelty that no virtual phantom‐based measurements for multiple‐target SRS plans have been published so far. A limitation of this study is that it did not include VMAT plans, because this technology was not available at HQB at the time of its preparation. However VIPER operation is identical for VMAT provided cine‐images are obtained with known gantry angles.

At this point, the question arises about the advantage of using the EPID measurements and reconstructing the dose in the VCP20 (VIPER software) compared to taking the Dynalog files and recomputing the delivered fields in the VCP20 using the PRIMO software. It is well described that the DynaLogs files can be used in combination with a dose calculation algorithm (e.g., MC simulation) to estimate the dose delivered to a patient.^31^, ^33^ Although these logs contain information of the MLC leaf positions for each delivered MU by the linac, they do not provide a direct and independent measurement of the actual fluence delivered or beam intensity, in contrast to the use of an EPID. Moreover it is reported that some MLC malfunctions are not recorded on the treatment logs.^48^, ^49^ However, they can be detected with an EPID acquisition. So, we think that a more independent check of the SRS plan can be done using EPID‐based 3D dose reconstruction instead of using a MC and Dynalog‐based procedure.

The MLC used in this study has 40 central leaf pairs of 5 mm width (covering the central 20 cm field size), and 20 outer leaf pairs of 1 cm width (covering up to 40 cm field size). Therefore, the 1 cm leaves could compromise the treatment of very small targets. According to our procedure for SIMT SRS, the treatment isocenter is located at the center of the brain such that it always lies in the 20‐cm field (superior‐inferior length) where only 5‐mm leaves are available. In this way, all lesions included in this study were covered by the 5‐mm leaves. According to our clinical routine, we have not found so far a brain with more than 20 cm as superior‐inferior length.

The VIPER verification of a SRS plan is based on a virtual phantom, that is, a real phantom is not used and therefore any fiducial or landmark is available to check the targeting accuracy. For instance, the isocenter wobble of the couch is not taken into account by VIPER, as the EPID is insensitive to the couch rotation.

In summary, this study has demonstrated that the VIPER software can be a streamlined alternative to commercially available 2D detector arrays, solving their drawbacks related to the detector field coverage and resolution when SIMT plans has to be verified. By contrast, the VIPER software has some limitations as: (a) 3D dose distribution calculation with respect to the patient’s CT anatomy cannot be done, as performed by other softwares;^34^, ^50^ (b) reconstruction of non‐coplanar fields onto the VCP20 phantom is not supported by the version of VIPER used in this study; and (c) VIPER cannot replace the geometric checks used in SRS QA to verify the isocenter and targeting accuracy, like a Winston‐Lutz test or an end‐to‐end test.^23^

The present study just assessed the feasibility of using the VIPER software for to be used as a tool for PSQA. To that purpose, the VIPER software needs to be validated independently of the TPS (with a MC simulation in this study).

## 5 CONCLUSIONS

VIPER software calculations were in agreement with PRIMO simulations within 5%/2 mm for clinical single‐isocenter multiple‐target SRS cases. Our results suggest using VIPER as a dosimetric check tool for pre‐treatment QA single‐isocenter multiple‐target plans. VIPER is an option to commercially available 2D detector arrays for this task.

## Conflict of interest

Dr Peter B. Greer is one of the VIPER developers. However, VIPER is not commercially available but it can be made available on request for research purposes. Rest of the authors have no relevant conflicts of interest to disclose.

## Author Contribution

The authors confirm contribution to the paper as follows:

Juan‐Francisco Calvo‐Ortega contributed to study conception and design.

Sandra Moragues‐Femenía and Coral Laosa‐Bello contributed to data collection.

Juan‐Francisco Calvo‐Ortega, Peter B. Greer, and Marcelino Hermida‐López contributed to analysis and interpretation of results.

Juan‐Francisco Calvo‐Ortega and Joan Casals‐Farran contributed to draft manuscript preparation.

All authors reviewed the results and approved the final version of the manuscript.
